# Pylephlebitis Following an Acute Angiocholitis: A Case Report

**DOI:** 10.7759/cureus.111091

**Published:** 2026-06-18

**Authors:** Mohamed Ali Nbaya, Wafa Guiza, Firas Kessentini, Yosri Amri, Imen Rejab

**Affiliations:** 1 Emergency Department, University Hospital of Gabes, Gabes, TUN

**Keywords:** abdominal emergency, acute cholangitis, biliary obstruction, point-of-care ultrasound (pocus), portal vein pylephlebitis, septic thrombosis

## Abstract

Acute cholangitis is a potentially life-threatening infection of the biliary tract that usually results from biliary obstruction, most commonly secondary to choledocholithiasis. Although prompt diagnosis and treatment often lead to favorable outcomes, uncommon complications such as pylephlebitis (septic thrombosis of the portal vein) may significantly increase morbidity and mortality.

We report the case of a 47-year-old woman with no significant past medical history who presented with right upper quadrant abdominal pain, fever (38.2°C), asthenia, and nausea. Physical examination revealed diffuse abdominal tenderness and mild jaundice. Abdominal point-of-care ultrasound (POCUS) made by the emergency physician on call showed multiple gallbladder calculi with sludge but without visible biliary dilatation. Laboratory evaluation demonstrated an inflammatory syndrome with C-reactive protein of 72 mg/L, normal leukocyte count, total bilirubin of 87 IU/L, and direct bilirubin of 56 IU/L, and cholestatic liver enzyme abnormalities, including alkaline phosphatase (ALP) of 190 IU/L and gamma-glutamyl transferase (GGT) of 108 IU/L. The diagnosis of acute angiocholitis was thus suspected. Abdominal imaging (computed tomography (CT) with contrast) showed intrahepatic and extrahepatic biliary dilatation caused by a distal common bile duct stone, multiple gallbladder calculi, and segment II left portal vein thrombosis consistent with pylephlebitis. A diagnosis of acute cholangitis secondary to choledocholithiasis complicated by pylephlebitis was established. The patient was treated with intravenous (IV) antibiotics, fluid resuscitation, and supportive care, with planned biliary decompression and definitive surgical management.

This case highlights a rare but serious vascular complication of biliary stone disease. Clinicians should maintain a high index of suspicion for portal venous involvement in patients with cholangitis and persistent systemic symptoms. Early imaging, timely antimicrobial therapy, and coordinated multidisciplinary management are essential to reduce the risk of severe complications and improve outcomes.

## Introduction

Acute cholangitis is a bacterial infection of the biliary tract that usually occurs secondary to biliary obstruction, most commonly caused by choledocholithiasis [1]. It remains a potentially life-threatening condition requiring prompt diagnosis and treatment to prevent sepsis and organ dysfunction. Although its presentation and management are well established, rare vascular complications may occur and worsen prognosis [2]. Early recognition through appropriate imaging and timely multidisciplinary management is essential for favorable outcomes. We report a case of acute cholangitis secondary to distal common bile duct stones complicated by segmental portal pylephlebitis.

## Case presentation

A 47-year-old woman presented to the emergency department with a one-week history of progressively worsening right upper quadrant abdominal pain associated with fever, nausea, and generalized weakness. Apart from allergic rhinitis every spring, the patient has no past medical and surgical history. She has no clotting disorders or autoimmune diseases. She had not visited the health center for any checkup in the past.

On admission and after receiving an intravenous (IV) drop of 1 g of paracetamol for pain, the patient was hemodynamically stable with a blood pressure of 110/60 mmHg, heart rate of 72 beats/minute, respiratory rate of 24 breaths/minute, and oxygen saturation of 98% on room air. Her temperature was 38.2°C. Cardiopulmonary examination was unremarkable. She was alert and oriented, with no focal neurological deficits. Abdominal examination revealed diffuse tenderness with guarding in the right upper quadrant. No palpable mass or signs of generalized peritonitis were noted. The patient had mild scleral icterus.

**Figure 1:**
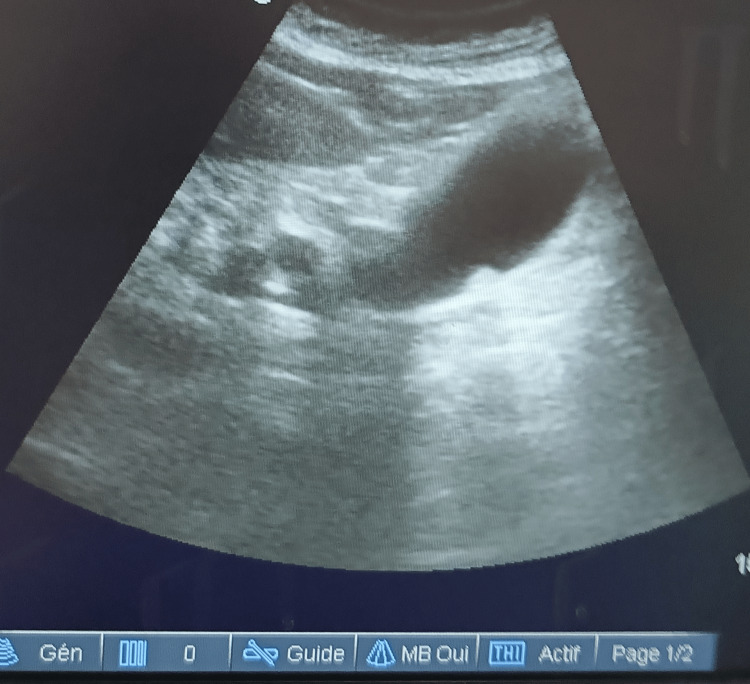
Abdominal POCUS showing multiple gallbladder calculi with sludge but without visible biliary distention POCUS: point-of-care ultrasound

Point-of-care ultrasound (POCUS) was performed and showed no intrahepatic and extrahepatic biliary dilation with gallbladder wall thickening measuring 9 mm. There was no visible collection around the gallbladder. The gallbladder was not distended, but gallstones and 02 calculi were visualized (Figure 1). Given the presence of fever, right upper quadrant pain, and jaundice consistent with Charcot’s triad, the diagnosis of acute angiocholitis was suspected along with the POCUS findings.

Initial laboratory investigations are summarized in Table 1.

**Table 1:** Initial laboratory investigations with normal range AST: aspartate transaminase, ALT: alanine transaminase, ALP: alkaline phosphatase, GGT: gamma-glutamyl transferase

Item	Value	Normal range
White blood cells	9,800/mm^3^	4,000-10,000/mm^3^
Hemoglobin	11.6 g/dL	12-17 g/dL
Platelets	385,000/mm^3^	150,000-400,000/mm^3^
C-reactive protein	72 mg/L	<6 mg/L
Prothrombin time	75%	>70%
Sodium	135.4 mmol/L	135-145 mmol/L
Potassium	3.54 mmol/L	3.5-4.5 mmol/L
Chlorine	99 mmol/L	95-105 mmol/L
Urea	2 mmol/L	2.8-7.2 mmol/L
Creatinine	26.9 μmol/L	58-104 μmol/L
AST	42.6 IU/L	4-50 IU/L
ALT	43.7 IU/L	4-50 IU/L
Total bilirubin	87 μmol/L	3-22 μmol/L
Direct bilirubin	56 μmol/L	<10 μmol/L
ALP	190 IU/L	30-120 IU/L
GGT	180 IU/L	<55 IU/L
Lipase	23 IU/L	<76 IU/L

**Figure 2:**
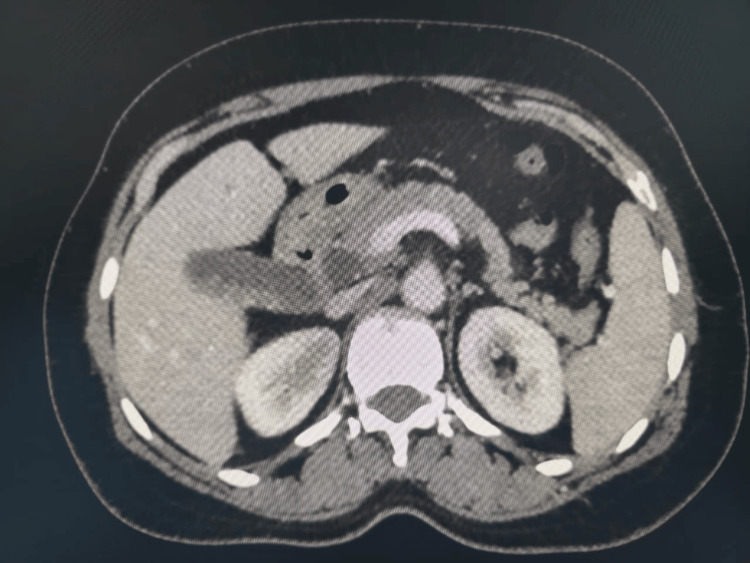
CT image showing dilatation of the extrahepatic biliary duct CT: computed tomography

**Figure 3:**
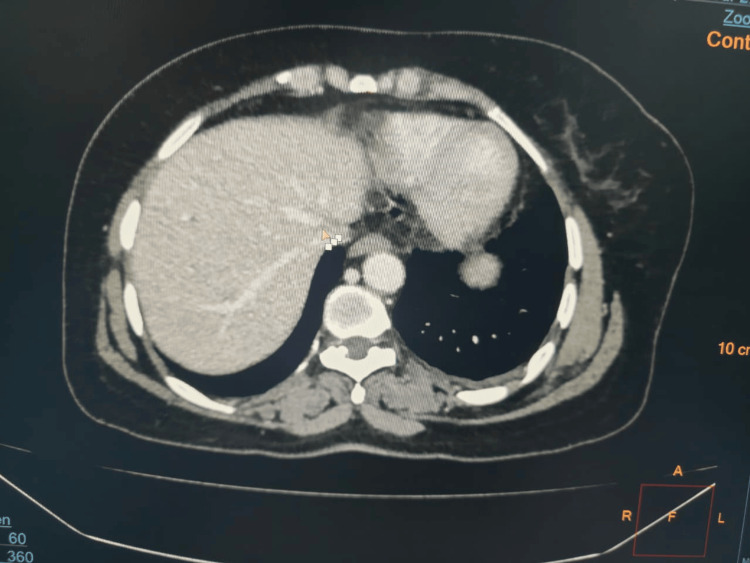
CT image showing an opacification defect of the portal vein CT: computed tomography

Contrast-enhanced abdominal computed tomography (CT) confirmed biliary tract dilation secondary to distal common bile duct lithiasis and findings consistent with acute cholangitis. In addition, thrombosis of the segment II branch of the left portal vein was identified, compatible with pylephlebitis (Figures 2, 3).

The patient was admitted to the surgical department and treated with intravenous fluids, analgesia, broad-spectrum intravenous antibiotics (cefotaxime, metronidazole, and gentamicin), and anticoagulation with low-molecular-weight heparin at a therapeutic dose (intramuscular injection 8,000 IU twice a day). Endoscopic retrograde cholangiopancreatography (ERCP) with biliary drainage was performed on hospital day 4 due to the lack of public ERCP centers in Tunisia (only one center in Mohamed Taher Maamouri Hospital in Nabeul). Cholecystectomy, as a final surgical management, was done on day 6.

The patient showed progressive clinical improvement following treatment and was discharged 10 days after the procedure in stable condition.

## Discussion

Pylephlebitis is an uncommon but potentially fatal septic thrombophlebitis of the portal venous system that develops as a complication of intra-abdominal infection [1]. It is classified into four anatomical groups [2,3]: group 1, thrombosis confined to the portal vein beyond the confluence of the splenic and superior mesenteric vein (SMV); group 2, extension of thrombus into the SMV but with patent mesenteric vessels; group 3, diffuse thrombosis of the splanchnic venous system but with large collaterals; and group 4, extensive splanchnic venous thrombosis but with only fine collaterals. Based on this classification, our patient had category 1 pylephlebitis.

It is considered a rare diagnosis with an estimated incidence ranging from 0.37 to 2.7 cases per 100,000 person-years [4,5]. A male predominance of approximately 70% has been reported in recent systematic reviews, along with a mean age of around 49 years [6,7]. Despite these observations, the condition is likely underrecognized because its clinical manifestations are nonspecific and frequently overlap with those of the primary infectious process.

The condition is characterized by suppurative thrombosis involving the portal vein or one of its tributaries following extension of local inflammation into the mesenteric venous circulation [7]. The portal venous system drains most of the digestive tract, including the stomach, small intestine, colon, pancreas, spleen, and portions of the hepatobiliary system through mesenteric and splenic tributaries. Consequently, pylephlebitis is classically described as a complication of gastrointestinal and intra-abdominal infections such as diverticulitis, appendicitis, liver abscess, and complicated enteric infections [4,8]. Among these, diverticulitis and appendicitis have historically been the most frequently reported etiologies [7].

The pathophysiology typically involves local venous inflammation in small draining veins adjacent to the septic focus, followed by endothelial injury, bacterial invasion, thrombus formation, and progressive extension toward larger portal venous branches [6]. This stepwise process explains how an initially localized infection may evolve into extensive portal venous thrombosis if not recognized early.

By contrast, pylephlebitis secondary to acute cholangitis or biliary lithiasis remains distinctly uncommon. This relative rarity can be explained by the fact that the biliary ducts are not components of the portal venous drainage system. Rather, they function as conduits for bile flow and are anatomically separate from venous structures, limiting direct propagation of infection into the portal circulation [1]. Furthermore, hepatic venous outflow occurs through the hepatic veins into the inferior vena cava, which is independent of portal inflow. As a result, infections originating in bowel segments that are directly drained by mesenteric tributaries are more likely to result in septic portal thrombosis than biliary infections [6].

Nevertheless, the close anatomical relationship between portal vein branches, hepatic arterial branches, and biliary radicals within the portal triads provides a plausible pathway for indirect inflammatory spread. Severe biliary infection may lead to periductal inflammation, endothelial injury, regional venous stasis, and secondary thrombosis of adjacent portal venous branches.

This mechanism may explain the localized thrombosis observed in our patient, involving the segment II branch of the left portal vein rather than the main portal trunk or the mesenteric circulation. Segmental involvement supports the hypothesis of contiguous regional inflammatory extension rather than embolic or systemic thrombotic disease. In biliary-associated cases, thrombosis may remain confined to intrahepatic portal branches and therefore be easily overlooked unless cross-sectional imaging is carefully reviewed [9].

The diagnosis of pylephlebitis remains challenging because symptoms are often nonspecific. Fever, abdominal pain, nausea, malaise, and elevated inflammatory markers may reflect either the primary infection or the thrombotic complication itself [6,10]. When present, jaundice and cholestatic liver test abnormalities may be attributed solely to cholangitis, potentially delaying recognition of portal venous involvement. Clinicians should therefore maintain a high index of suspicion in patients with intra-abdominal infection who exhibit persistent fever, bacteremia, worsening abdominal pain, or delayed clinical improvement despite appropriate therapy [11].

In the present case, imaging performed to evaluate suspected acute cholangitis also revealed portal branch thrombosis, allowing early diagnosis before the development of more severe complications. Contrast-enhanced computed tomography is considered the preferred imaging modality, as it can simultaneously identify the infectious source and detect venous thrombosis [9]. Typical findings include filling defects within the portal vein or its branches, along with surrounding inflammatory changes or associated intra-abdominal pathology.

Doppler ultrasonography may also demonstrate portal venous thrombosis as heterogeneous echogenic material within the vessel lumen; however, it is operator-dependent and may be limited by patient-related factors such as bowel gas or obesity [9,12]. Magnetic resonance imaging may be useful in selected cases but is less frequently employed in acute settings.

Microbiologically, pylephlebitis is frequently polymicrobial and reflects enteric flora. Commonly isolated organisms include *Escherichia coli*, *Bacteroides fragilis*, *Klebsiella* species, *Streptococcus* species, and anaerobes [8,13]. Rare pathogens such as *Listeria monocytogenes* have also been reported [14]. Blood cultures may be positive but are not universally so, and negative results do not exclude the diagnosis [6]. For this reason, empiric antibiotic therapy should provide broad-spectrum coverage against both aerobic and anaerobic organisms and should later be tailored according to microbiological findings when available [13].

Management requires both eradication of infection and control of thrombus progression [6]. Prolonged intravenous broad-spectrum antibiotic therapy remains the cornerstone of treatment [15]. In addition, effective source control is essential and depends on the underlying etiology [16]. In biliary-associated cases, prompt decompression of the obstructed biliary tree is fundamental [7,15].

In our patient, endoscopic retrograde cholangiopancreatography with biliary drainage was performed after initial management, which likely contributed significantly to the favorable clinical outcome. The delay in performing ERCP was due to the lack of ERCP centers among public health hospitals in Tunisia, as only one center is available, and it is located in Mohamed Taher Maamouri Hospital in Nabeul in the North of Tunisia.

The role of anticoagulation in pylephlebitis remains controversial, as current evidence is based primarily on retrospective studies and case reports rather than randomized trials [6,17]. Nevertheless, anticoagulation is frequently considered in cases with thrombus extension, mesenteric vein involvement, persistent fever despite antibiotic therapy, underlying hypercoagulable conditions, or progression on follow-up imaging [18]. Potential benefits include prevention of thrombus propagation, promotion of recanalization, and reduction of long-term complications such as portal hypertension.

Various anticoagulant agents have been used, including low-molecular-weight heparin, unfractionated heparin, and direct oral anticoagulants targeting factor Xa or thrombin [10,18,19]. Thrombolytic therapy is not routinely recommended, although isolated cases of successful catheter-directed thrombolysis have been reported [20]. In this case, anticoagulation with low-molecular-weight heparin was initiated due to documented portal branch thrombosis and was associated with a favorable clinical evolution.

If left untreated, pylephlebitis can lead to severe complications, including hepatic abscess formation, septic embolization, bowel ischemia, cavernomatous transformation of the portal vein, chronic portal hypertension, and death [5]. Although mortality rates were historically high, outcomes have improved significantly with advances in imaging, early diagnosis, and multidisciplinary management [13]. Prognosis ultimately depends on the timeliness of diagnosis, the extent of thrombosis, the virulence of the underlying infection, and the adequacy of source control.

## Conclusions

This case is clinically noteworthy because it illustrates an atypical origin of pylephlebitis. While most cases arise from infections of bowel segments drained directly by the portal tributaries, acute cholangitis can also lead to portal venous septic thrombosis through regional inflammatory extension within the hepatoduodenal and intrahepatic portal triad structures. Recognition of this possibility is important because portal branch thrombosis may be subtle and easily missed when attention is focused primarily on biliary obstruction. In patients presenting with acute cholangitis, especially those with persistent fever, severe inflammatory response, or unusual imaging findings, clinicians should consider associated portal venous complications. Early cross-sectional imaging and coordinated management involving emergency physicians, radiologists, gastroenterologists, surgeons, and internists can significantly improve outcomes.
